# Inference of Gene Regulatory Network Based on Local Bayesian Networks

**DOI:** 10.1371/journal.pcbi.1005024

**Published:** 2016-08-01

**Authors:** Fei Liu, Shao-Wu Zhang, Wei-Feng Guo, Ze-Gang Wei, Luonan Chen

**Affiliations:** 1 Key Laboratory of Information Fusion Technology of Ministry of Education, School of Automation, Northwestern Polytechnical University, Xi’an, China; 2 Institute of Physics and Optoelectronics Technology, Baoji University of Arts and Science, Baoji, China; 3 Key Laboratory of Systems Biology, Innovation Center for Cell Signaling Network, Institute of Biochemistry and Cell Biology, Shanghai Institutes for Biological Sciences, Chinese Academy of Sciences, Shanghai, China; 4 School of Life Science and Technology, ShanghaiTech University, Shanghai, China; University of Calgary Cumming School of Medicine, CANADA

## Abstract

The inference of gene regulatory networks (GRNs) from expression data can mine the direct regulations among genes and gain deep insights into biological processes at a network level. During past decades, numerous computational approaches have been introduced for inferring the GRNs. However, many of them still suffer from various problems, e.g., Bayesian network (BN) methods cannot handle large-scale networks due to their high computational complexity, while information theory-based methods cannot identify the directions of regulatory interactions and also suffer from false positive/negative problems. To overcome the limitations, in this work we present a novel algorithm, namely local Bayesian network (LBN), to infer GRNs from gene expression data by using the network decomposition strategy and false-positive edge elimination scheme. Specifically, LBN algorithm first uses conditional mutual information (CMI) to construct an initial network or GRN, which is decomposed into a number of local networks or GRNs. Then, BN method is employed to generate a series of local BNs by selecting the *k*-nearest neighbors of each gene as its candidate regulatory genes, which significantly reduces the exponential search space from all possible GRN structures. Integrating these local BNs forms a tentative network or GRN by performing CMI, which reduces redundant regulations in the GRN and thus alleviates the false positive problem. The final network or GRN can be obtained by iteratively performing CMI and local BN on the tentative network. In the iterative process, the false or redundant regulations are gradually removed. When tested on the benchmark GRN datasets from DREAM challenge as well as the SOS DNA repair network in *E*.*coli*, our results suggest that LBN outperforms other state-of-the-art methods (ARACNE, GENIE3 and NARROMI) significantly, with more accurate and robust performance. In particular, the decomposition strategy with local Bayesian networks not only effectively reduce the computational cost of BN due to much smaller sizes of local GRNs, but also identify the directions of the regulations.

## Author Summary

Gene regulatory network (GRN) represents how some genes encode regulatory molecules such as transcription factors or microRNAs for regulating the expression of other genes. Accurate inference of GRN is an important task to understand the biological activity from signal emulsion to metabolic dynamics, prioritize potential drug targets of various diseases, devise effective therapeutics, and discover the novel pathways. In this paper, we propose a novel local Bayesian network (LBN) algorithm to improve the accuracy of GRN inference from gene expression data by exploring advantages of Bayesian network (BN) and conditional mutual information (CMI) methods. BNs with kNN network decomposition and CMI are respectively introduced to reduce the high computational complexity of BN and remove the false or redundant regulation interactions. The superior performance of the proposed LBN approach is demonstrated on GRN datasets from DREAM challenge as well as the SOS DNA repair network in *E*. *coli*.

## Introduction

Gene regulatory networks (GRNs) that explicitly characterize regulatory processes in cells are typically modeled by graphs, in which the nodes represent the genes and the edges reflect the regulatory or interaction relationship between genes [1]. Accurately inferring GRN is of great importance and also an essential task to understand the biological activity from signal emulsion to metabolic dynamics, prioritize potential drug targets of various diseases, devise effective therapeutics, and discover the novel pathways [2–4]. Identifying the GRNs with experimental methods is usually time-consuming, tedious and expensive, and sometimes lack of reproducibility. In addition, recent high-throughput sequencing technologies have yielded a mass of gene expression data [5], which provides opportunity for understanding the underlying regulatory mechanism based on the data. Therefore, numerous computational approaches have been developed to infer the GRNs [3, 6–45]. Such computational methods can be roughly categorized into the co-expression based approaches [6], supervised learning-based approaches [7–13], model-based approaches [3, 14–30], and information theory-based approaches [31–40]. The co-expression based methods have low computational complexity, but they cannot infer direct associations or model system dynamics. The supervised learning-based methods make use of the known regulations to infer GRNs on a genome-wide data, such as SEREND [8], GENIES [9] and SIRENE [11], but require additional information of the regulatory interactions to train a model. By guiding the inference engine from the prior information of the known regulations, it can achieve higher precision and outperform many other methods [46]. However, the insufficient information of the labeled or known gene datasets limits the application of this kind of approaches [47, 48].

On the other hand, model-based methods can be further classified into ordinary differential equation [14, 15], multiple linear regression [18, 19], linear programming [20, 21], Boolean networks [17, 22], and probabilistic graphical models including Bayesian network (BN) [3, 16, 23, 49] and graphical Gaussian model [24, 25]. Overall, these model-based methods can provide us a deeper understanding of the system’s behaviors at a network level and can also infer the directions of regulations in the network. However, these methods are parameters-dependent and time-consuming, which makes them difficult to deal with large-scale networks. For example, inferring GRNs based on the probabilistic graphical models requires to search the optimal graph from all possible graphs with respect to all genes in the network. Due to this NP-hard nature [50] of learning static Bayesian network structure, two common alternative techniques, i.e., a heuristic-based search [26] and the maximum-number-of-parents (maxP) [27, 28] were developed approximately to search the sub-optimal graphs. Yet, the heuristic search approaches still have high computational complexity and do not guarantee global optimal. Although the maxP technique by limiting the maximum number of parents for each gene to *q* can partly reduce the computational complexity, it needs to traverse search all genes for inferring the parents of one gene. Thus, maxP techniques have polynomial complexity of *O*(*n*^*q*^) for an *n*-node GRN [28], which are still unsuitable for large-scale GRNs. To reconstruct dynamic Bayesian networks (DBNs), two structure learning algorithms such as BNFinder [29] and globalMIT [30] have been proposed to infer GRNs, but these algorithms are currently suitable only for small networks since they also require to search all combinations of regulators for a gene.

Recently, information theory-based methods are widely used for reconstructing GNRs, such as mutual information (MI) [33, 34–36, 42–44] and conditional mutual information (CMI) [31, 38, 45]. These approaches are assumption-free methods, measuring unknown, non-linear and complex associations rather than linear-correlations between genes [38, 40], and addressing the problem of intense computation for parameters. Thus, they can be used to infer large-scale GRNs. However, MI-based methods overestimate the regulation relationships to some extent and fail to distinguish indirect regulators from direct ones, thereby leading to possible false positives [38, 51, 52]. Although CMI-based methods are able to separate the direct regulations from the indirect ones, they cannot derive the directions of regulations in the network and also tend to underestimate the regulation strength in some cases [32, 37, 45].

**Fig 1:**
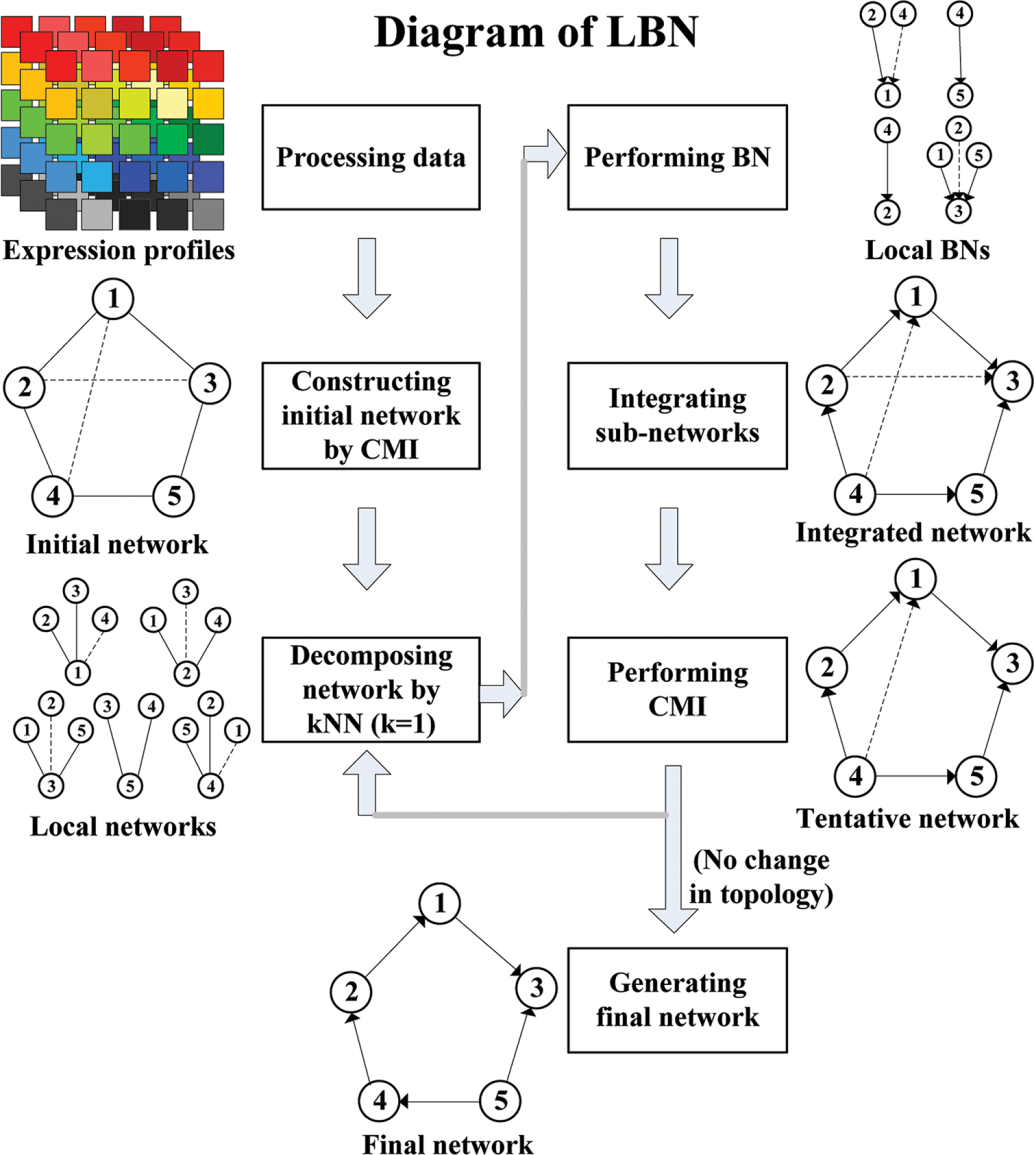
Schematic diagram of LBN method. (1) process the data, (2) construct the initial network (a large-scale network) by CMI or MI, (3) decompose the network into local networks (a number of small-scale networks) by *k*NN with *k* = 1, (4) perform BN to obtain local BNs (a number of small-scale networks), (5) integrate local BNs into a candidate network (a large-scale network), (6) perform CMI to obtain the tentative network (a large-scale network). Iteratively performing BN and CMI with *k*NN (*k* = 2) until *G*_*C*_ topological structure tends to stable, the final network or GRN can be inferred. The solid lines denote the true regulations and the dashed lines denote redundant correlations between two genes.

To overcome these limitations of BN, MI and CMI, in this paper, we propose a novel local Bayesian network (LBN) algorithm to reconstruct GRNs from gene expression data by making use of their advantages, i.e., infer the directed network with less false-positive edges and with high computational efficiency. LBN algorithm mainly consists of five distinct elements shown in Fig 1: i) CMI is first employed to construct an initial network, i.e., *G*_*MI*_, which then is decomposed into a series of smaller sub-networks, i.e., local networks or GRNs, according to the nearest relationship among genes in the network with *k*-nearest neighbor (kNN) method. ii) For these local networks or GRNs, BN method is used to identify their regulatory relationships with directions, generating a series of local BNs which are integrated into a candidate GRN *G*_*B*_. iii) CMI is applied to remove the false positive edges in *G*_*B*_, forming a tentative GRN *G*_*C*_. iv) According to the relationships of kNN among genes in the network, the tentative GRN (*G*_*C*_) is further decomposed into a series of smaller sub-networks or local networks, in which BN method is implemented to delete the false regulatory relationships. v) The final network or GRN *G*_*F*_ is inferred by iteratively performing BN and CMI with kNN decomposition until the topological structure of the tentative network *G*_*C*_ does not change. On the benchmark GRN datasets from DREAM challenge [53, 54] and widely used SOS DNA repair network in Escherichia coli [55, 56], the simulation results confirmed the effectiveness of our LBN algorithm, which is superior to other three state-of-the-art approaches, i.e., ARACNE [36], GENIE3 [13] and NARROMI [20].

## Results and Discussion

### Datasets and evaluation metrics

The benchmark network datasets play an important role in assessing the effectiveness of algorithms in reconstructing GRNs. Many researchers used the simulated datasets derived from DREAM challenge [53] to evaluate their algorithms. DREAM challenge gives a series of gene expression datasets with noise and gold benchmark networks, which were selected from source networks of real species. In this work, we utilized three simulation datasets as well as two real gene expression datasets to validate our method. The three synthetic datasets in sizes 10, 50 and 100 (marked as dataset10, dataset50 and dataset100, respectively) obtained from DREAM3 challenge contain 10, 50 and 100 genes with 10, 77 and 125 edges, respectively, which come from 10, 50 and 100 samples respectively. The real gene expression dataset is the well-known SOS DNA repair network with experiment dataset in *E*. *coli *[55, 56], which includes 9 genes with 24 edges. Another large-scale gene expression dataset from *E*. *coli* data bank [57] is an experimentally verified network [58], which includes 1418 genes with 2675 edges.

In order to validate our algorithm, the true positive rate (*TPR*), false positive rate (*FPR*), false discovery rate (*FDR*), positive predictive value (*PPV*), overall accuracy (*ACC*), *F*-score measure and Matthews correlation coefficient (*MCC*) are used to evaluate the performance of our LBN and other algorithms. These metrics are defined as follows:

$$\begin{array}{l} TPR=TP/(TP+FN),\;FPR=FP/(FP+TN),\\ FDR=FP/(TP+FP),\;PPV=TP/(TP+FP),\\ ACC=(TP+TN)/(TP+FP+TN+FN),\\ F=2PPV\times TPR/(PPV+TPR),\\ MCC=\frac{TP\times TN-FP\times FN}{\sqrt{\left(TP+FP)(TP+FN)(TN+FP)(TN+FN\right)}} \end{array}$$ (1)

where *TP* is the number of edges that are correctly identified, *TN* is the number of non-link edges correctly identified, *FP* is the number of edges that are incorrectly identified, *FN* is the number of non-link edges incorrectly identified. By setting different *CMI* threshold values varying from large to small with a fixed scale, we obtained a series of *TP*, *FP*, *TN* and *FN* to calculate their corresponding *TPR* and *FPR* values, which are used to plot the receiver operating characteristic (ROC) curves. The area under ROC curves (AUC) is calculated as another metric for comparing different algorithms.

### Evaluating simulation datasets

Three synthetic datasets (dataset10, dataset50 and dataset100) from DREAM3 challenge were used to assess LBN algorithm, and three state-of-the-art methods of GENIE3 [13], ARACNE [36], and NARROMI [20] were chosen to evaluate the performance of LBN and those methods. GENIE3 [13] decomposes the problem of inferring a regulatory network of *p* genes into *p* different feature selection problems by using Random Forest and Extra-Trees algorithms. ARACNE [36] utilizes the data processing inequality to eliminate the majority of indirect interactions inferred by co-expression methods, which cannot recover all transcriptional interactions in a GRN but rather to recover some transcriptional interactions with a high confidence. NARROMI [20] combines the information theory-based CMI and the path-consistent algorithm (PCA) to improve the accuracy of GRN inference. In NARROMI, MI is firstly used to remove the noisy regulations with low pairwise correlations, and then CMI is utilized to exclude the redundant regulations from indirect regulators iteratively by PCA from a lower order to a higher order. For all the methods in comparison, the parameters were set to default values.

We use the Z-statistic test [59, 60, 38] at the significance level of *P-value* = 0.05 to select the suitable thresholds for parameters *α* and *β*, which are approximately *α* = 0.03, 0.1 and 0.1 as the threshold values of CMI to construct the gene correlation network *G*_*MI*_ for dataset10, dataset50 and dataset100 respectively. In the same way, we also selected *β* = 0.03, 0.1 and 0.1 as the threshold value of CMI to remove the false positive edges for dataset10, dataset50 and dataset100 respectively. The results in Table 1 show that our LBN method has the highest PPV, ACC, MCC, F and AUC scores among all, except that the AUC of ARACNE on the dataset 100 is a little higher than that of our LBN method. The results on the three datasets with different network sizes selected from real and experimental verified networks in *Yeast* genomes also demonstrate the effectiveness of our LBN in terms of higher and more robust performances in inferring GRNs.

**Table 1:** Comparison of different methods on dataset10, dataset50 and dataset100.

Method	TPR	FPR	FDR	PPV	ACC	MCC	F	AUC
**Dataset10**								
GENIE3	0.700	0.112	0.563	0.437	0.867	0.483	0.538	0.919
ARACNE	**0.900**	0.112	0.500	0.500	0.888	0.618	0.643	0.930
NARROMI	0.700	0.050	0.364	0.636	0.922	0.623	0.666	0.938
LBN	**0.900**	**0.050**	**0.308**	**0.692**	**0.944**	**0.759**	**0.782**	**0.942**
**Dataset50**								
GENIE3	0.481	0.078	0.833	0.167	0.908	0.245	0.248	0.843
ARACNE	**0.597**	0.082	0.809	0.192	0.908	0.303	0.291	0.832
NARROMI	0.532	0.062	0.783	0.217	0.925	0.307	0.308	0.839
LBN	0.403	**0.011**	**0.456**	**0.544**	**0.971**	**0.453**	**0.463**	**0.863**
**Dataset100**								
GENIE3	0.265	0.015	0.768	0.232	0.972	0.234	0.247	0.809
ARACNE	**0.421**	0.042	0.854	0.146	0.949	0.227	0.217	**0.887**
NARROMI	0.277	0.010	0.676	0.324	0.978	0.289	0.299	0.849
LBN	0.283	**0.005**	**0.510**	**0.490**	**0.983**	**0.364**	**0.359**	0.852

In addition, there are a number of methods for inferring GRNs based on Markov Blanket, such as Grow-shring [61], IAMB [62] and Fast-IAMB [63]. Both of Grow-shring and IAMB methods first identify the Markov Blankets for each variable (or node) by iteratively executing a series of conditional independence and dependence tests, then connect nodes in a consistent way to infer Bayesian network. However, in the process of discovering the Markov Blanket of a target variable T, Grow-shring and IAMB methods require to search almost all other variables, which increases algorithm’s time complexity. Although the computational complexity (O(n^2^)) of these two methods is in the same scale as our method and is lower than that of BN method (O(2^n^)), numerical computations show that our method performs superior to them for simulation dataset and real datasets or large-scale GRNs. Specifically, in order to assess effectiveness of our LBN method, we compared LBN with Grow-shring and IAMB methods on dataset10. The comparative results of three methods are shown on Table 2, from which we can see that the computational time of our LBN method is considerably lower than that of either Grow-shring method or IAMB method. In addition, as shown in Table 2, the accuracy of our GRN inference is also high.

**Table 2:** Comparison of Grow-shring, IAMB and LBN methods on dataset10.

Method	TPR	FPR	FDR	PPV	ACC	MCC	F	Runtime(s)
Grow-shring	0.700	0.100	0.533	0.467	0.878	0.506	0.560	128.815
IAMB	0.800	0.075	0.429	0.571	0.911	0.629	0.667	70.524
LBN	0.900	0.050	0.308	0.692	0.944	0.759	0.782	10.462

### Inferring SOS network and gene regulatory interactions in *E*. *coli*

**Fig 2:**
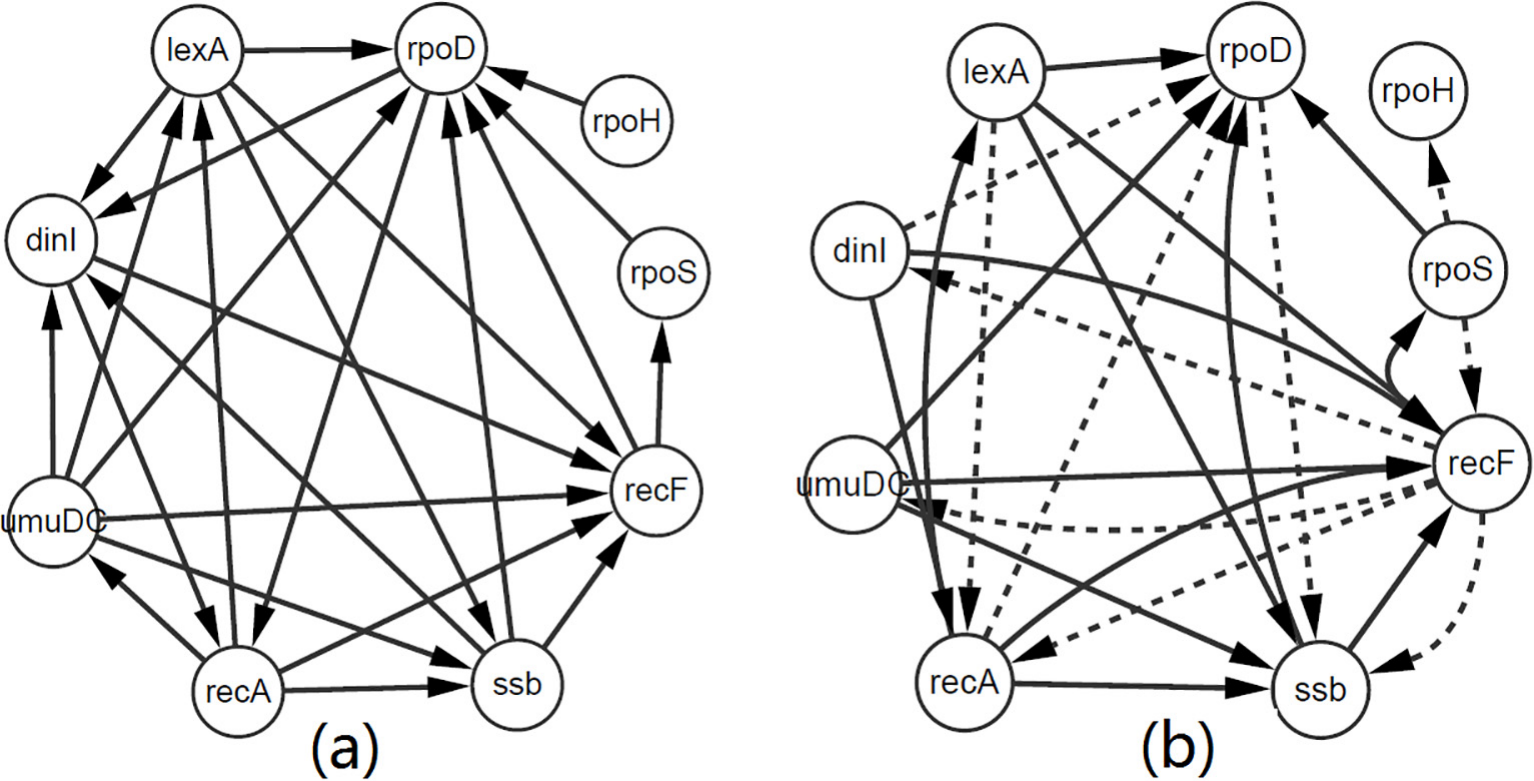
SOS DNA repair network. (a) True network. (b) Inferred network with LBN (*α* = *β* = 0.01). The solid lines are correctly inferred regulatory relationships, and the dotted lines are false regulatory links.

In order to further evaluate the performance of our LBN algorithm, we also implemented our LBN method and other five methods, i.e., GENIE3, ARACNE,NARROMI, Grow-shring and IAMB on the well-known SOS DNA repair network, which is an experimentally verified network in *E*. *coli*, with real gene expression data [55, 56]. SOS network (Fig 2A) includes two mediators of the SOS response (lexA and recA), four other regulatory genes (ssb, recF, dinI and umuDC) involved in the SOS response, and three sigma factor genes (rpoD, rpoH and rpoS) whose regulations play important roles in the SOS response. Choosing threshold *α* = *β* = 0.01, the comparison results of LBN with GENIE3, ARACNE, NARROMI, Grow-shring and IAMB are shown on Table 3, in which we can see that the performance of our LBN method is also superior to GENIE3, ARACNE, NARROMI, Grow-shring and IAMB. For example, the ACC of LBN is 73.6%, which is 4.2%, 25%, 15.3%, 9.7% and 2.8% higher than that of GENIE3, ARACNE, NARROMI, Grow-shring and IAMB, respectively, and AUC of LBN achieves at 0.816, which is 0.132, 0.077, 0.025, 0.058 and 0.007 higher than that of GENIE3, ARACNE, NARROMI, Grow-shring and IAMB, respectively. Fig 2B gives the SOS network inferred with LBN, which shows that LBN method infers 15 true regulatory relationships and 10 false regulatory links. These results also indicate that our LBN method can infer most of the true regulatory relationships between genes, and verify the effectiveness and efficiency of LBN method on the real gene expression data.

**Table 3:** Comparison of different methods on SOS DNA repair network.

Method	TPR	FPR	FDR	PPV	ACC	MCC	F	AUC
GENIE3	0.500	**0.208**	0.455	0.546	0.694	0.299	0.522	0.684
ARACNE	**0.708**	0.625	0.638	0.362	0.486	0.083	0.479	0.739
NARROMI	0.667	0.458	0.579	0.421	0.583	0.197	0.516	0.791
Grow-shring	0.458	0.271	0.542	0.458	0.639	0.188	0.458	0.758
IAMB	0.583	0.229	0.440	0.560	0.708	0.351	0.571	0.809
LBN	0.625	**0.208**	**0.400**	**0.600**	**0.736**	**0.412**	**0.612**	**0.816**

LBN was also applied to construct a large-scale GRN from real gene expression data. We used the experimentally verified reference network in *E*. *coli* [58] to evaluate the performance of LBN, and downloaded the gene expression data from the well-known *E*. *coli* data bank [57]. The experimentally verified network includes 2675 edges between 160 regulators and 1258 targets that can be found in the gene expression dataset [20]. The comparison results of LBN with GENIE3, ARACNE, NARROMI, Grow-shring and IAMB on the large-scale gene regulatory network in *E*. *coli* are listed on Table 4, from which we can see that the proposed LBN method performs better than other methods with the highest average AUC scores, number and proportion for regulators and target genes. These results indicate that our LBN method is also suitable to infer large-scale GRNs.

**Table 4:** Comparison of different methods on the large-scale gene regulatory network.

	GENIE3	ARACNE	NARROMI	Grow-shring	IAMB	LBN
AveAUC_TF	0.684	0.749	0.754	0.724	0.751	0.761
#AUC>0.7(rate)	78(0.486)	86(0.538)	93(0.581)	84(0.525)	89(0.556)	96(0.600)
#AUC>0.8(rate)	60(0.375)	68(0.425)	71(0.444)	62(0.389)	68(0.425)	72(0.450)
AveAUC_TG	0.723	0.733	0.735	673(0.535)	690(0.548)	0.747
#AUC>0.7(rate)	484(0.385)	691(0.549)	694(0.552)	472(0.375)	479(0.381)	702(0.558)
#AUC>0.8(rate)	428(0.340)	484(0.385)	485(0.386)	602.776	472.598	488(0.388)

Notes: AUC represents the area under ROC curve; AveAUC_TF is average AUC for transcriptional factors (TFs); AveAUC_TG is average AUC for target genes (TGs);

#**(rate) is the number and proportion of TFs/TGs predicted correctly under the condition **.

### Effects of the strategies of network decomposition and false-positive edge deletion

**Fig 3:**
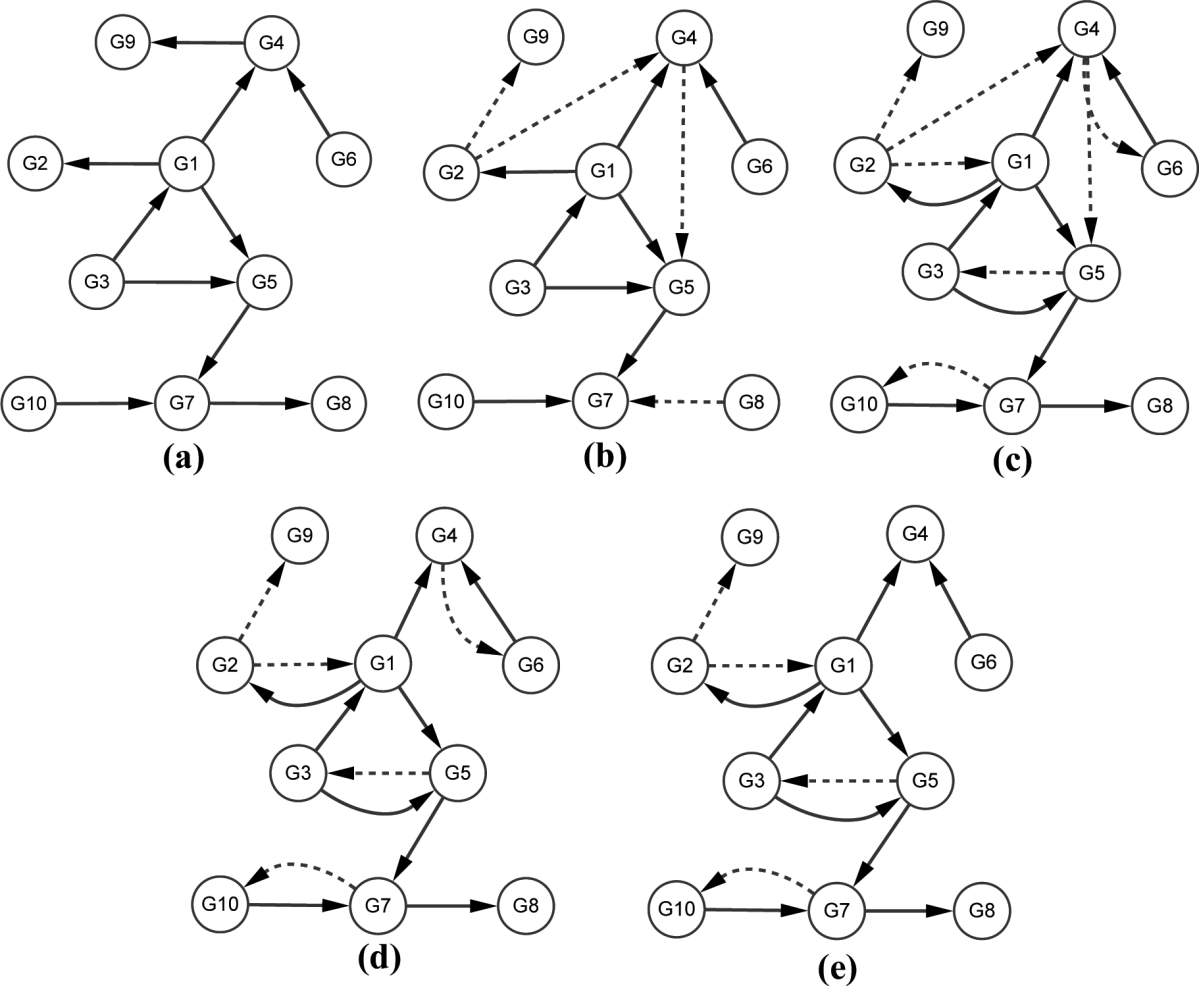
Gene regulatory networks composed of 10 genes. (a) The true network with 10 genes and 10 edges. (b) The network inferred by BN method. (c) The network inferred by MI+BN. (d) The network inferred by MI+BN+CMI. (e) The network inferred by MI+BN+CMI+kNN+BN. The solid lines are correctly inferred regulatory relationships, and the dotted lines are false regulatory links.

In order to evaluate the effectiveness of the strategies of the network decomposition and false-positive edge deletion introduced in our LBN algorithm, we tested the performance of different combination ways (i.e. MI+BN, MI+BN+CMI, MI+BN+CMI+kNN+BN) on the dataset10, which includes 10 genes and 10 regulatory edges. MI+BN denotes that MI method was firstly used to construct the initial GRN, then the network decomposition strategy and BN method were adopted to generate GRN; MI+BN+CMI denotes that MI, the network decomposition strategy and BN method were used to infer GRN, then CMI was chosen to remove the false positive edges; MI+BN+CMI+kNN+BN denotes that MI, the network decomposition strategy, CMI and BN methods were used to generate GRN, then kNN and BN methods were respectively taken to decompose GRN, reconstruct GRN and further delete the false positive edges. On the same PC (i5-2400 CPU, 4GB RAM), the results of different combination ways were listed on Table 5. Fig 3 shows the true gene regulatory network (a) that was selected from an experimental verified network in *Yeast* genome, the inferred networks (b), (c), (d) and (e) generated by BN, MI+BN, MI+BN+CMI and MI+BN+CMI+kNN+BN, respectively. From Table 5 and Fig 3, we can see that the running time of MI+BN was 0.7852s lower than that of BN, while it wrongly predicted 7 regulatory edges, which means that the strategy of MI+BN effectively reduces the computational time, meanwhile it results in more false positive edges; CMI can really remove the false positive edges, and kNN indeed helps the Bayesian network accurately learning and reducing the false positive edges. These results indicate that our strategy of the network decomposition can significantly reduce the high computation cost of the BN method for large-scale GRNs, whereas the strategy of deleting the false-positive edges with CMI and kNN can remarkably enhance the accuracy of the network inference.

**Table 5:** Results of different combination ways on the dataset10.

Method	TPR	FPR	FDR	PPV	ACC	MCCC	F	Time (s)
BN	0.800	0.050	0.333	0.667	0.933	0.693	0.727	0.8247
MI+BN	0.900	0.088	0.438	0.563	0.911	0.668	0.692	0.0395
MI+BN+CMI	0.900	0.063	0.357	0.643	0.933	0.726	0.750	0.0544
MI+BN+CMI+kNN+BN	0.900	0.050	0.308	0.692	0.944	0.759	0.782	0.2677

### Effects of the threshold parameters

**Fig 4:**
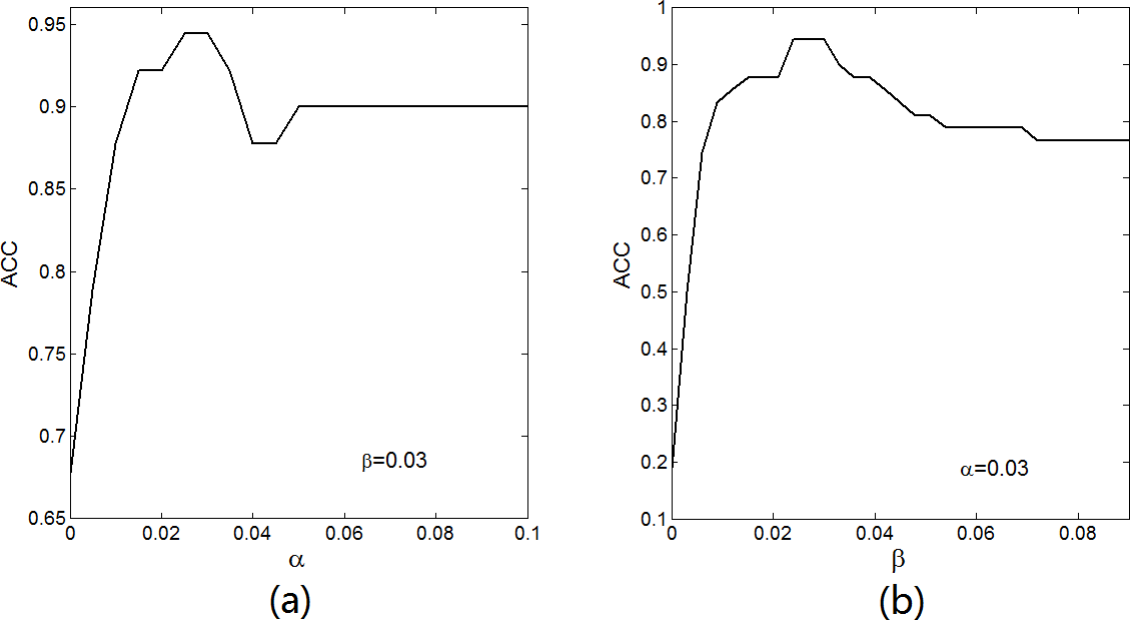
Effect of parameters *α* and *β *for LBN on dataset10.

There are two parameters *α* and *β* in our LBN algorithm, which determine whether or not there is a link or an edge between two genes in the reconstructed GRN. In order to evaluate the impact of *α* and *β* parameters in LBN algorithm, we performed simulations on dataset10 by calculating ACC with different *α* and *β* values by fixing another parameter, and the simulated results are shown in Fig 4. From Fig 4A, we found that the ACC value increases gradually in the range 0 ≤ *α* < 0.025, and reaches the highest value (ACC = 0.944) in the range 0.025 ≤ *α* ≤ 0.03, and decreases gradually in the range 0.03 < *α* < 0.045, while the ACC basically remains unchanged (*ACC* ≈ 0.9). From Fig 4B, we found that the ACC value increases gradually in the range 0 ≤ *β* < 0.024, and reaches the highest value (ACC = 0.944) in the range of 0.024 ≤ *β* ≤ 0.03, and decreases gradually in the range 0.03 < *β* < 0.09. Although the parameters α and β have some influence on the results of the inferred GRNs, the effect is minor in those threshold ranges. Thus, we can select *α* and *β* lied in these range (e.g., 0.025 ≤ *α* ≤ 0.03 and 0.024 ≤ *β* ≤ 0.03) to obtain the best GRN for dataset10. We also performed simulations by calculating ACC with different α and β values on dataset50, dataset100 and SOS DNA dataset, respectively. The experimental results show that we should select the suitable parameters (*α* and *β*) for different datasets to obtain the best GRNs.

### Analysis of LBN computational complexity

The computational complexity of LBN method involves five phases or parts. In the phase of inferring an initial network, LBN needs to compute MI or CMI value of each gene pair at zero order, thus the maximum complexity is in the order of *O*(*n*^*2*^), where *n* is the total number of genes. For the phase of constructing the directed network, LBN needs to select regulatory genes for each target gene, and thus the maximum complexity is in the order of *O*(*n*×*2*^*m*^), where *m* is the number of regulatory genes, and *m* << *n*. In the phase of filtering false positive edges by CMI, the time complexity is *O*(*n*^*2*^). For the phase of further removing the redundant edges with kNN, LBN needs to find *n* sub-networks, and hence the time complexity is *O*(*n*). In last phase of iteratively performing CMI and BN with kNN methods until the topological structure of the tentative or candidate network does not change. If iteratively performing *l* times, then the total complexity of LBN is *O*(2*l*×*n*^*2*^*+l*×*n+l*×*n*×2^*m*^). When *n* is very large and *m *<< *n*, the computational complexity of LBN is *O*(*n*^*2*^), which is lower than that of BN method (*O*(2^*n*^)).

### Conclusions

In this work, we presented a novel method, namely LBN, to improve the accuracy of GRN inference from gene expression data by adopting two strategies, i.e., the network decomposition and the false-positive edge deletion, which can accurately infer a directed network with high computational efficiency. Specifically, the network decomposition can effectively reduce the high computational cost of BN method for inferring large-scale GRNs, whereas CMI with kNN can delete the redundant regulations and thus reduce the false positives. By iteratively performing CMI and BN with kNN methods, LBN algorithm can infer the optimal GRN structure with regulation directions. The results on the benchmark gene regulatory networks from the DREAM3 challenge and a real SOS DNA repair network in *E*. *coli* show that our LBN method outperforms significantly other three state-of-the-art methods of ARACNE, GENIE3 and NARROMI. Clearly, our LBN makes Bayesian network accurately to learn the network structure and reduce the false positives by searching *k*-nearest neighbors of every gene, and thus, LBN is effective and robust for inferring the directed GRNs. On the other hand, based on probabilistic graphical model, a network inference method called the module network method [64] was also developed. Compared with Segal’s Module network method [64] which infers the network among modules, our LBN algorithm adopts the iterative algorithm between CMI and probabilistic graphical model (i.e., BN) to infer the network among genes.

Despite the above advantages of LBN, it can be improved from the following two aspects. Firstly, it is still a challenging task to select the parent genes of *X* gene in the set of variables, which will affect the computational cost and accuracy of inferring GRNs. Secondly, the inferred network is a static network, and thus it is a future direction to extend LBN to consider the dynamical features in the network, e.g., Dynamic Bayesian Networks (DBNs) or Dynamical Network Markers (DNMs) [65] by using time-course or stage-course data, which can be found in wider applications [66–68] in biomedical fields.

## Methods

### MI and CMI

Recently, both of mutual information (MI) and conditional mutual information (CMI) have been widely applied to inferring GRNs [20, 31, 38, 40, 55, 56, 69], due to their capability of characterizing nonlinear dependency, which provides a natural generalization of association between genes. MI can be used to measure the degree of independence between two genes *X*_*i*_ and *X*_*j*_, but it tends to overestimate the regulation strengths between genes (i.e., false positive problem). On the other hand, CMI measures the conditional dependency between two genes *X*_*i*_ and *X*_*j*_ given other gene *X*_*k*_, which can quantify the undirected regulation.

For discrete variables *X* and *Y*, MI is defined as [31, 38, 70, 71]:

$$MI(X,Y)=-\sum_{x\in X,y\in Y}p(x,y)\;\text{log}\frac{p(x,y)}{p(x)p(y)}=H(X)+H(Y)-H(X,Y)$$ (2)

where *p*(*x*, *y*) is the joint probability distribution of *X* and *Y*, and *p*(*x*) and *p*(*y*) are the marginal probability distributions of *X* and *Y*, respectively; *H*(*X*) and *H*(*Y*) are the entropies of *X* and *Y*, respectively; and *H*(*X*,*Y*) is the joint entropy of *X* and *Y*.

CMI between two variables *X* and *Y* given variable *Z* is defined as [31, 38, 70]:

$$CMI(X,Y|Z)=\sum_{x\in X,y\in Y,z\in Z}p(x,y,z)\;\text{log}\frac{p(x,y|z)}{p(x|z)p(y|z)}=H(X,Z)+H(Y,Z)-H(Z)-H(X,Y,Z)$$ (3)

where *p*(*x*,*y|z*), *p*(*x|z*) and *p*(*y|z*) are conditional probability distributions, and *H*(*X*, *Z*), *H*(*Y*, *Z*), and *H*(*X*, *Y*, *Z*) are the joint entropies.

With the widely adopted hypothesis of Gaussian distribution for gene expression data, the entropy can be estimated by the following Gaussian kernel probability density function [38, 42],

$$P(X_{i})=\frac{1}{N}\sum_{j=1}^{N}\frac{1}{{\left(2\pi \right)}^{n/2}{\left|C\right|}^{n/2}}\text{exp}(-\frac{1}{2}{\left(X_{j}-X_{i}\right)}^{T}C^{-1}(X_{j}-X_{i}))$$ (4)

where *C* is the covariance matrix of variable *X*, |*C*| is the determinant of the matrix, *N* is the number of samples and *n* is the number of variables (genes) in *C*. Generally, if the sample number is almost equal to the gene number, the empirical covariance matrix is often used to estimate the covariance matrix of the distribution of gene expression profile, which can be considered as a good approximation of the true covariance matrix. However, when the number of samples is smaller than that of genes, the regularized covariance matrix [72, 73] is used to estimate the covariance matrix of the distribution of gene expression profile. The number of replicate samples will affect the performance of the method, and increasing replicate samples can enhance the GRN inference algorithm’s power.

Thus, the entropy of variable *X* can be denoted as:

$$H(X)=\text{log}[{\left(2\pi e\right)}^{n/2}{\left|C\right|}^{1/2}]=\frac{1}{2}\text{log}[{\left(2\pi e\right)}^{n}\left|C\right|]$$ (5)

According to Eqs 2 and 5, MI between two variables (genes) *X* and *Y* can be easily calculated by using the following equivalent formula [31, 38, 70].

$$MI(X,Y)=\frac{1}{2}\text{log}\frac{\left|C(X)\right|•\left|C(Y)\right|}{\left|C(X,Y)\right|}\;.$$ (6)

High *MI* value indicates that there may be a close relationship between the variables (genes) *X* and *Y*, while low MI value implies their independence. If variables (genes) *X* and *Y* are independent of each other, clearly *MI*(*X*, *Y*) = 0.

Similarly, under the assumption of Gaussian distributions for gene expression data, CMI of two variables (genes) *X* and *Y* given variable (gene) *Z* can be easily calculated by using the following equivalent formula [31, 38].

$$CMI(X,Y|Z)=\frac{1}{2}\text{log}\frac{\left|C(X,Z)\right|•\left|C(Y,Z)\right|}{\left|C(Z)\right|•\left|C(X,Y,Z)\right|}.$$ (7)

Obviously, when *X* and *Y* are conditionally independent given *Z*, *CMI*(*X*, *Y*|*Z*) = 0. In addition, this equivalent expression is an efficient method to calculate CMI between two variables *X* and *Y* given one or more variables *Z*, e.g., if the conditional variable *Z* = (*Z*_1_, *Z*_2_) is composed of two variables *Z*_1_ and *Z*_2_, we can obtain the second-order CMI.

### Bayesian networks

A Bayesian network (BN) is a graphical model of the probabilistic relationships among a set of random variable *X* = {*X*_1_,*X*_2_,…,*X*_*i*_,….*X*_*n*_}, which is a directed acyclic graph *G*. In a Bayesian network, the vertices (nodes) are the random variables (genes), and the edges represent the probabilistic dependencies among the corresponding random variables (genes). Under the Markov assumption that given its parents, each variable is independent of its non-descendants, the relationships between the variables (genes) are described by a joint probability distribution *P*(*X*_1_,*X*_2_,…,*X*_*n*_), which can be decomposed into a product of conditional probabilities based on the graphical structure:

$$P(X_{1},X_{2},...,X_{n})=\prod_{X_{i}\in X}P(X_{i}|Pa(X_{i}))$$ (8)

where *Pa*(*X*_*i*_) is the set of parents of node *X*_*i*_ in graph *G*.

In the process of BN structure learning, the most likely graph *G* for a given dataset *D* can be inferred by searching for the optimal graph based on a Bayesian scoring metric. That is, by trying out all possible graphs *G* (i.e., all possible combinations of interaction among genes), the graph *G* with the maximum Bayesian score (joint probability) is chosen as the most likely gene regulatory network. In general, the number of possible graph *G* grows exponentially with the number of nodes (or genes), and the problem of identifying the optimal graph is NP-hard [50]. For a larger dataset *D* which contains more variables, it is not computationally possible to calculate the Bayesian score for all possible graphs *G*. Therefore, the heuristic search methods, such as greedy-hill climbing approach, the Markov Chain Monte Carlo method and simulated annealing, are often used to infer the Bayesian network structure [28, 74].

Here, the optimal graph *G* can be decomposed into a series of optimal sub-graphs, each of which is centered on one node or gene. However, the parent set of every node *X*_*i*_ may be consisted of other nodes in *G*, the computational complexity of identifying the optimal sub-graphs is considerably high, i.e., it is still not computationally possible to calculate the maximum Bayesian score of all possible sub-graphs of every node for a large-scale network. Generally, the neighbor genes of gene *X*_*i*_ most likely regulate it. Thus, we limit the size of parents of each node *X*_*i*_ to approximately calculate the maximum Bayesian score of every node.

In this paper, as shown in Fig 1, we first construct the undirected network with CMI method, and decompose the network into a series of sub-networks in which the central node just is linked with its *k* nearest neighbors (or nodes). Due to every sub-network just contains a few nodes, we can identify the set of parents of every central node by calculating the Bayesian scores of all possible sub-network structures of the central node to choose the optimal Bayesian sub-network with maximum joint probability distribution score. Then, by integrating all of the sub-networks, we have the candidate global Bayesian network (or GRN). Note that BN can be extended to dynamic Bayesian network by using time-course expression data.

### *k*-nearest neighbor

In a graph *G*(*V*,*E*), *V* represents a set of nodes and *E* represents edges between nodes. The *k* closest neighbors of each node are selected according their shortest path distance in the graph structure. That is, the *k*-nearest neighbor (kNN) of node *V*_*i*_ consists of a set of nodes whose shortest path to the node *V*_*i*_ is *k*. In this paper, we used the *k*-nearest neighbors of each node to decompose a large-scale network to form a series of local Bayesian networks. For each local Bayesian network, the Bayesian network inference method is used to remove the false positive edges. For a large-scale network, we show that it can actually achieve a high accuracy even with the first- and second-nearest neighbors of each node. Actually, the *k*-nearest neighbors of a gene or node with *k* = 2 contains the Markov blanket of the node, which includes all the *k*-nearest neighbors with *k* = 1 and the partial the *k*-nearest neighbors with *k* = 2 for that node. The Markov blanket of a node in a Bayesian network is composed of all the variables that shield the node from the rest of the network, which implies that the Markov blanket of a node is the only knowledge needed to predict the behavior of that node. Thus, we choose *k* = 2 in this paper.

### LBN algorithm

Given an expression dataset with *n* genes and *N* samples, a novel algorithm (called LBN) was developed to infer its underlying GRN. As shown in Fig 1, LBN is composed of four main parts: i) Construct an initial network (or GRN) with MI or CMI method, ii) Decompose the large-scale (initial) network into series of sub-networks by *k*NN method, i.e., local networks or GRNs, iii) Identify the regulatory relationship among genes by BN method for each sub-network, and iv) Integrate all local BNs as a candidate network, and then remove the false regulatory relationships by CMI, i.e., construct the tentative network. Then we have the final network or GRN by iteratively performing CMI and BN with kNN methods. Numerical computations show that our LBN method can infer the final GRN after iterating 10–20 times. Fig 1 is the schematic diagram of our LBN method, which is described in detail as follows:

#### Step 1: Construct the initial network by CMI

In general, the gene-gene pairs with high MI or CMI values are co-expressed genes, in which one is the target gene, and another is the regulatory gene (regulator). For an expression dataset with *n* genes, we first compute the MI or CMI values between all gene pairs with Eq 6, deleting these edges whose MI values are smaller than a pre-defined threshold *α*, and then construct an initial GRN which is an undirected network *G*_*MI*_.

#### Step 2: Decompose *G*_*MI*_ into *n* sub-networks or local networks by kNN

For a larger network *G*_*MI*_ which contains a large number of genes, it is a NP-hard problem to try out all its possible structures to search for the most likely gene regulatory network with BN method. Therefore, we proposed a strategy to bypass this problem by decomposing network *G*_*MI*_ into series of sub-networks which contains a few genes. Suppose every gene *g*_*i*_ in the network *G*_*MI*_ is a potential target gene, and its nearest neighbor genes in *G*_*MI*_ are its potential regulatory genes (regulators), that is, gene *g*_*i*_ and its nearest neighbor genes form a local network *G*_*MI*_. Based on this assumption, the network *G*_*MI*_ can be decomposed into *n* sub-networks or local networks, where *n* is the total number of genes in the network. Every sub-network is composed of the gene *g*_*i*_ and its nearest neighbor genes.

#### Step 3: Construct local BNs by estimating the gene regulations and integrate local BNs into a candidate network

For every sub-network, we calculate the joint probability distribution value of all its possible structure, selecting the network with the maximum joint probability distribution value as the optimal Bayesian sub-network from which we can identify the candidate regulatory genes (regulators) of the target *g*_*i*_. Then, the *n* optimal Bayesian sub-networks or local BNs are integrated into a directed network *G*_*B*_ as a candidate network or GRN from which we can find the regulatory relationship between genes. In the process of constructing the Bayesian sub-network, it can not only identify the edge direction between the interacting genes, but also eliminate the redundant regulation edges.

#### Step 4: Construct tentative network by eliminating the redundant regulations by CMI

MI method commonly tends to overestimate the regulation strengths between genes, which does not consider the joint regulations of a target gene by other two or more genes, and thus results in more false positive edges. In this step, we use CMI to remove false positive edges by computing the first-order *CMI*(*i*, *j*|*k*), second-order *CMI*(*i*, *j*|*k*, *l*) with Eq 7. If *CMI*(*i*, *j*|*k*) (or *CMI*(*i*, *j*|*k*, *l*)) is smaller than a pre-defined threshold *β*, the edge linked genes *i* and *j* is deleted from network *G*_*B*_. Thus, we can generate a tentative network or GRN *G*_*C*_.

#### Step 5: Decompose *G*_*C*_ into *N* smaller networks or local networks

In Steps 2 and 3, the sub-networks decomposed from *G*_*MI*_ are the smallest local networks whose shortest path is 1 (i.e., *k* = 1). Using these sub-networks to construct local GRN with BN method may introduce some false regulatory edges. For further filtering the false genes regulatory edges, we should enlarge the parent set of each gene. However, if selecting more neighbors for one gene as its candidate regulators, it will increase the computational complexity. In this work, we select *k* = 2 to enlarge the parent set of each gene. Thus, we applied the second-nearest neighbor of each node to decompose *G*_*C*_, forming *n* sub-networks whose shortest path is 2 (i.e., *k* = 2), then using the BN method to reconstruct local GRNs for every sub-networks. The candidate GRN *G*_*C*_ is calculated by iteratively performing Steps 3–5 until its topological structure does not change. In the end, we can obtain the final network or GRN *G*_*F*_.
